# Effect of liver resection-induced increases in hepatic venous pressure gradient on development of postoperative acute kidney injury

**DOI:** 10.1186/s12882-021-02658-7

**Published:** 2022-01-08

**Authors:** Christian Reiterer, Alexander Taschner, Florian Luf, Manfred Hecking, Dietmar Tamandl, Oliver Zotti, Thomas Reiberger, Patrick Starlinger, Mattias Mandorfer, Edith Fleischmann

**Affiliations:** 1 Department of Anaesthesia, Intensive Care Medicine and Pain Medicine, Medical University of Vienna, Spitalgasse 23, 1090 Vienna, Austria; 2 Outcomes Research Consortium, Cleveland, OH USA; 3 Department of Anaesthesia and Intensive Care, Hanusch Krankenhaus, Vienna, Austria; 4 Division of Nephrology and Dialysis, Dept. of Internal Medicine III, Medical University of Vienna, Vienna, Austria; 5 Department of Biomedical Imaging and Image-guided Therapy, Medical University of Vienna, Vienna, Austria; 6 Division of Gastroenterology and Hepatology, Dept. of Internal Medicine III, Medical University of Vienna, Vienna, Austria; 7 Christian Doppler Laboratory for Portal Hypertension and Liver Fibrosis, Medical University of Vienna, Vienna, Austria; 8 Department of Surgery, Medical University of Vienna, Vienna, Austria

**Keywords:** Acute kidney injury, Liver resection, Hepatic venous pressure gradient, Renin-angiotensin-aldosterone system

## Abstract

**Background:**

The impact of changes in portal pressure before and after liver resection (defined as ΔHVPG) on postoperative kidney function remains unknown. Therefore, we investigated the effect of ΔHVPG on (i) the incidence of postoperative AKI and (ii) the renin-angiotensin system (RAAS) and sympathetic nervous system (SNS) activity.

**Methods:**

We included 30 patients undergoing partial liver resection. Our primary outcome was postoperative AKI according to KDIGO criteria. For our secondary outcome we assessed the plasma renin, aldosterone, noradrenaline, adrenaline, dopamine and vasopressin concentrations prior and 2 h after induction of anaesthesia, on the first and fifth postoperative day. HVPG was measured prior and immediately after liver resection.

**Results:**

ΔHVPG could be measured in 21 patients with 12 patients HVPG showing increases in HVPG (∆HVPG≥1 mmHg) while 9 patients remained stable. AKI developed in 7/12 of patients with increasing HVPG, but only in 2/9 of patients with stable ΔHVPG (*p* = 0.302). Noradrenalin levels were significantly higher in patients with increasing ΔHVPG than in patients with stable ΔHVPG. (*p* = 0.009). Biomarkers reflecting RAAS and SNS activity remained similar in patients with increasing vs. stable ΔHVPG.

**Conclusions:**

Patients with increased HVPG had higher postoperative creatinine concentrations, however, the incidence of AKI was similar between patients with increased versus stable HVPG.

## Introduction

Acute kidney injury (AKI) occurs in about 6% of patients undergoing noncardiac surgery [1]. Moreover, after liver surgery the incidence reaches up to 15%.^1^ The underlying pathophysiology of the high incidence of AKI in patients undergoing liver-resection is still not fully understood. A possible explanation might be the effect of liver resection on the intrahepatic vascular resistance (IVR). A postoperative increase of IVR might lead to an “*acute hepatorenal like syndrome”* affecting kidney function due to the increased release of endogenous vasopressors resulting in kidney arterial vasoconstriction [2–4].

Increased portal vein pressure caused by liver resection [5, 6] leads to splanchnic vasodilation and further reduce systemic vascular resistance [7]. This results in an activation of the sympathetic nervous and the renin-angiotensin system, in order to maintain an adequate organ perfusion pressure [8]. Furthermore, the activation of the neurohumoral system results in an excessive release of noradrenaline and angiotensin II [8]. These pathophysiologic mechanisms impair kidney perfusion and reduce kidney function [8].

In the non-operative setting, measurement of the hepatic venous pressure gradient (HVPG) is an established method to stratify liver pathophysiology in patients with cirrhosis [9]. However, there is scarce evidence regarding the impact HVPG changes in the perioperative period, specifically in patients without underlying cirrhosis undergoing liver resection. The difference between preoperative measured HVPG and postoperative measured HVPG during liver resection; which we defined as ΔHVPG.

In order to explain the higher incidence of AKI after liver resection, we tested the effect of ΔHVPG on the incidence AKI as our primary outcome. We further evaluated the effect of ΔHVPG on the activity of sympathetic nervous system and renin-angiotensin system as our secondary outcome.

## Methods

This study was approved by the Institutional Review Board of the Medical University of Vienna (EK 424/2010) and registered at ClinicalTrials.gov (NCT01700231). We obtained written informed consent from all patients prior to enrollment. The study was conducted according to the “Declaration of Helsinki” and followed the ICH GCP Guidelines at the Medical University of Vienna. We included patients between 18 and 85 years with neoplastic liver tumors undergoing elective hepatic resection. In detail, patients underwent liver resection because of known hepatocellular carcinoma, cholangiocarcinoma or liver metastases. Patients with a preoperative *HVPG* > 10 mmHg and kidney failure were excluded. Specifically, only patients with chronic kidney disease stage four or five according to the National Kidney Foundation (NKF) (defined by glomerular filtration rate < 30 mL/min) were excluded.

### Patient characteristics and recorded parameters

Demographic data including age, sex, BMI, American Society of Anesthesiologists (ASA) physical status, comorbidities, type of surgery and preoperative laboratory values were recorded. We recorded routine intraoperative variables including time of anesthesia and surgery, and fluid and anesthesia management. We recorded esophageal doppler derived hemodynamic data including stroke volume and cardiac output. Intraoperative core temperature was measured and the distal esophagus.

Laboratory liver and kidney function tests included: serum-creatinine, serum-albumin, prothrombin index and bilirubin. These were assessed before surgery and on the postoperative days 1 and 5. Postoperative maximum concentration was defined as the maximum value measured within 2 h after surgery, on the first and fifth postoperative day.

Acute kidney injury was diagnosed according to the Kidney Disease: Improving Global Outcome (KDIGO) definitions based on serum creatinine levels [10], considering the maximum increase during the first 48 h postoperative days (PODs): ∆ Cr = Maximum (Cr_POD1_, Cr_POD2_) / Cr_Preop_.

### Anaesthesia protocol

For induction of anaesthesia 3–4 mg/kg propofol, 2–3 μg/kg fentanyl and 0.6 mg/kg rocuronium was given. Anaesthesia was maintained with sevoflurane in 30% oxygen carrier. We administered additional boli of fentanyl or rocuronium according to the patient’s requirements. Ventilatory rate was adjusted to maintain an end-tidal P_CO2_ of 35–40 mmHg. Normothermia was maintained with forced air warming. Standard monitoring included electrocardiography (ECG), invasive blood pressure and pulse oximetry. A central venous catheter was inserted after induction of anesthesia. Mean arterial pressure was maintained above 65 mmHg. We used an esophageal doppler (Cardiac Q, Deltex Medical Group PLC, Chichester, UK) for goal-directed fluid therapy according to a previously published protocol [11]. All patients were given 5–7 mL kg^− 1^ of lactated Ringer solution during induction of anaesthesia and followed by 3–5 mL kg^− 1^ per hour normalized to ideal body weight for maintenance throughout surgery. Red cells were transfused as necessary to maintain a hematocrit level above 26%.

### Liver resection surgery

Surgery was performed according to standardized operational protocol. We used a cavitron ultrasound surgical aspiration (Integra CUSA Excel) for liver parenchyma dissection. To coagulate the small vessels we used bipolar coagulation, while major vascular structures were ligated or sewed. The weight of the resected liver tissue was recorded.

### Invasive hemodynamic measurements

Portal pressure is clinically evaluated by measuring the hepatic venous pressure gradient (HVPG), i.e. the difference between the wedged hepatic venous pressure and the free hepatic venous pressure, as previously described [9]. Briefly, a catheter introducer sheet is placed in the jugular vein using the Seldinger technique, and a main hepatic vein was cannulated under fluoroscopic guidance using a 7F balloon-tipped catheter (Straight Occlusion Catheter, Medi-Tech; Boston Scientific Cork Ltd., Cork, Ireland). After fluoroscopic control of a sufficient wedge position in the hepatic vein, we measured free and wedged hepatic venous pressures in triplicates to calculate the HVPG. The inferior vena cava pressure was also recorded to rule out an increased post-hepatic pressure. HVPG was measured preoperatively after induction of anesthesia and immediately after end of surgery before extubation. ∆HVPG was defined as difference between the pre- and the postoperative HVPG. Patients were stratified according to the *∆HVPG* into two groups as (i) *∆HVPG ≥ 1 mmHg,* defined as postoperative HVPG higher than preoperative HVPG and (ii) ΔHVPG ≤0 mmHg defined as postoperative HVPG was similar or lower than preoperative HVPG.

### Statistics

Statistical analysis was performed with IBM SPSS Statistics (Version 25). Patient characteristics, demographic data, preoperative laboratory values, kidney and liver specific data were compared for balance. Normal distribution of data was tested using a Kolmogorov-Smirnov test. Normally distributed data were presented as mean ± standard deviation, not normally distributed data were given as median and percentile. Chi-square test was used to comparing categorical variables.

We stratified patients into two groups: patients with ∆HVPG *≥1 mmHg* and patients with stable ∆ HVPG. We compared the incidence of AKI between both groups using a Fisher exact test. Fluid management and hemodynamics were compared between both groups using a Student’s test or a Mann-Whitney-U test as appropriate.

To determine the effect of ΔHVPG on the postoperative sympathetic nervous system and renin-angiotensin system activity we measured renin, aldosterone, noradrenalin, adrenalin, dopamine and vasopressin concentrations before, 2 h after induction of anaesthesia, and on the first and fifth postoperative day. Postoperative maximum concentrations of renin, angiotensin, dopamine, noradrenalin and adrenaline were compared between the two groups using Mann-Whitney-U test.

To investigate, whether a change in HVPG is an independent predictor for AKI, we further compared renin, aldosterone, noradrenalin, adrenalin, dopamine and vasopressin concentrations as well as liver volumetrics between patients with and without AKI using Mann-Whitney-U tests.

Postoperative maximum concentrations of liver and kidney function tests including creatinine, albumin, prothrombin index and bilirubin were compared between both groups using a Mann-Whitney-U test. Variables were summarized as median [25th, 75th percentiles] or mean (SD). Categorical variables were summarized as frequency (percent). We used SPSS for statistical analysis. (Version 25, IBM SPSS Statistic, Armonk, NY, USA).

## Results

We included 30 patients undergoing elective liver resection from October 2010 to July 2013 at the Medical University of Vienna. We were unable to measure the postoperative hepatic venous pressure in 9 patients. Therefore, these patients were excluded from the final analysis. Baseline characteristics and laboratory measurements were shown in the online supplements.

Baseline characteristics, demographics, comorbidities, type of surgery, and laboratory values were similar between both groups. (Table 1).

**Table 1:** Baseline characteristics

		∆HVPG ≥1 mmHg (*n* = 12)		∆HVPG ≤0 mmHg (*n* = 9)		*p* - Value
Age, *yrs*		73	[62, 79]	70	[52, 73]	0.027
Height, *cm*		172	(7)	171	(11)	0.841
Weight, kg		74	(13)	79	(18)	0.458
Sex, no. (*%*)						
	Men	2	(17)	6	(67)	0.375
	Woman	10	(83)	3	(33)	
Comorbidities, no. (*%*)						
	Arterial Hypertension	5	(42)	2	(29)	0.373
	NIDDM	1	(0)	1	(14)	0.667
	Pulmonary	0	(0)	2	(29)	0.076
	NASH	4	(33)	2	(29)	0.488
Type of Surgery, no. (*%*)						0.005
	Hemihepatectomy	10	(83)	2	(22)	
	Partial Liver Resection	2	(17)	7	(78)	
Laboratory Parameters						
	Bilirubin, *mg/dL*	1	[0.7; 1.4]	0.5	[0.4; 0.7]	0.002
	ASAT, *U/L*	49	[23; 82]	44	[24; 28]	0.336
	ALAT, *U/L*	35	[21; 61]	27	[14; 45]	0.546
	Creatinine, *mg/dL*	0.8	(0.2)	0.9	(0.1)	0.819
	CRP, *mg/dL*	0.2	[0.1; 0.3]	0.7	[0.1; 0.8]	0.722
	Albumin, *g/L*	34.0	(3)	36.0	(4)	0.728
	Prothrombin Time, %	73	(11)	87	(16)	0.259
	Cholinesterase, *kU/L*	4.9	(1.0)	6.8	(1.5)	0.108
	Platelets, G/dL	137	(37)	190	(46)	0.018
	vWF, %	176	[114; 200]	186	[159; 285]	1.000
	RiCo, %	154	(49)	153	(61)	0.606
	Fibrinogen, *mg/dL*	300	(68)	348	(146)	0.163

Summary statistics of patient characteristics are presented as counts, percentages of patients, means (±SD), and median [25th percentile, 75th percentile]. All *P*-values are for unpaired Student’s-*t* tests, Mann-Whitney-U test or chi-square tests as appropriate. *NIDDM, non-insulin dependent diabetes mellitus; NASH; non-alcoholic steatohepatitis; ASAT, aspartate aminotransaminase; ALAT, alanine aminotransaminase; CRP, C-reactive protein; vWF, von Willebrand factor; RiCo, Ristocetin cofactor*

In 12 patients ∆HVPG was ≥1 mmHg and in 9 patients ∆HVPG remained unchanged (∆HVPG ≤0). There was no difference in the incidence of AKI according to the KDIGO criteria between patients with ∆HVPG ≥1 mmHg as compared to patients with unchanged ∆HVPG ≤0 mmHg (*p* = 0.302). (Table 2) Postoperative maximum creatinine was significantly higher in patients with ∆HVPG ≥1 mmHg as compared to those with ∆HVPG ≤0 mmHg. (1.4 mg/dL [1.2; 1.7] and 1.1 mg/dL [1.2; 1.6]; *p* = 0.039) (Table 2).

**Table 2:** Outcome parameters

		∆HVPG ≥1 mmHg (n = 12)		∆HVPG ≤0 mmHg (n = 9)		*p -* Value
*Hepatic Venous Pressure Gradient*						
Preoperative, *mmHg*		3.5	[2.0; 6.8]	4.0	[2.0; 7.0]	1.000
Postoperative, *mmHg*		6.5	[4.0; 9.8]	4.0	[2.0; 6.5]	0.041
∆HVPG, *mmHg*		2.0	[1.0; 3.8]	0.0	[−1.0; 0.0]	< 0.001
**Primary Outcome**						
*KDIGO, no. (%)*						0.302
None		5	(42)	7	(78)	
I		4	(33)	2	(22)	
II		2	(17)	0	(0)	
III		1	(8)	0	(0)	
Max Creatinine, *mg/dL*		1.4	[1.2; 1.7]	1.1	[1.2; 1.6]	0.039
**Secondary Outcomes**						
*Renin-angiotensin-aldosterone-system*						
Renin, μlE*/mL*	Baseline	7.8	[2.2; 62.0]	51.3	[0.6; 63]	1.000
	Max	29.5	[8.6; 155.4]	85.6	[2.1; 296.6|	0.833
Aldosterone, *pg/mL*	Baseline	25	[15; 170]	53	[35; 103]	0463
	Max	169	[28; 273]	130	[8; 253]	0.970
Vasopressin, *pg/dL*	Baseline	1.2	[< 1; 11.2]	< 1	[< 1; < 1]	0.021
	Max	7.6	[2.0; 45.0]	12.7	[3.4; 18.1]	0.930
*Sympathetic Nervous System*						
Noradrenalin, *ng/dL*	Baseline	276	[213; 2431]	218	[113; 354]	0.200
	Max	3534	[2379; 19,679]	1376	[803; 2953]	0.009
Adrenalin, *ng/dL*	Baseline	13	[5; 62]	17	[5; 46]	0.595
	Max	258	[66; 443]	129	[85; 443]	0.568
Dopamine, *ng/dL*	Baseline	0	[0; 13]	5	[0; 23]	0.653
	Max	54	[43; 134]	62	[50;69]	0.653

Differences in the incidence of AKI stratified according to the KDIGO criteria are presented as counts and percentages of patients. Summary catecholamines and RAAS specific biomarkers are presented as medians [25th percentile, 75th percentile]. *p-*values are for chi-square or Mann-Whitney-U test as appropriate. *KDIGO, kidney disease improving global outcomes;*

Time of surgery and anesthesia, anaesthetic medication, intraoperative total fluid volume, urine output and blood loss did not differ between both groups. (Table 3) Intraoperative hemodynamic data were similar between both groups.

**Table 3:** Intraoperative characteristics

		∆HVPG ≥1 mmHg (n = 12)		∆HVPG ≤0 mmHg (*n* = 9)	*p* - Value	
Duration						
	Anaesthesia, *min*	300	[275; 360]	210	[181; 263]	0.046
	Surgery, *min*	240	[213; 264]	142	[125; 241]	0.019
Medication						
	Fentanyl, *μg*	850	[540; 1100]	750	[530; 825]	0.332
	Propofol, *mg*	225	[200; 250]	200	[200; 240]	0.520
	Metronidazol, *mg*	1500	[1500; 1500]	1500	[0; 1500]	0.304
	Cefuroxim, *mg*	1500	[1500; 1500]	1500	[1500; 1500]	0.868
Fluid Management						
	Total Fluid, *mL*	3331	(692)	2756	(641)	0.066
	Urine Output, *mL*	557	(417)	669	(408)	0.543
	Blood Loss, *mL*	500	[50; 775]	50	[0; 175]	0.070
Hemodynamic						
	MAP, *mmHg*	73	(4)	74	(7)	0.471
	SV, *mL*	66	(15)	70	(15)	0.625
	CO, *mL/min*	4.3	(1.0)	5.4	(1.6)	0.067
	CVP, *mmHg*	10	(3)	10	(3)	0.836
	Noradrenalin, *mg*	0.04	[0.03; 0.11]	0.028	[0.00; 0.04]	0.046
	Phenylephrine, *mg*	0	[0.0; 0.2]	0.2	[0.0; 0.63]	0.248
CT Liver Volumetrics						
	Pre-OP Liver Volume, *mL*	1640	[1385; 3119]	1564	[1270; 1793]	0.374
	Resected Liver Volume, *mL*	574	[195; 787]	84	[64; 179]	0.026
	Post-OP Liver Volume, *mL*	897	[706; 1255]	1361	[1137;1735]	0.003
	Tumor Volume, *mL*	177	[24; 1313]	12	[5; 62]	0.033

Summary characteristics of intraoperative measurements presented as means (SD) or medians [25th percentile, 75th percentile]. All *P*-values are for unpaired Student’s-*t* tests or Mann-Whitney-U tests as appropriate. *MAP, mean arterial pressure; SV, stroke volume; CO, cardiac output; CVP, central venous pressure; CT, computer tomography;*

Differences in liver specific laboratory parameters are shown in Table 4.

**Table 4:** Postoperative maximum liver specific parameters

	∆HVPG ≥1 mmHg (n = 12)		∆HVPG ≤0 mmHg (n = 9)		*p -* Value
Bilirubin, *mg/dL*	2.4	[1.4; 2.8]	1.2	[0.8; 1.2]	0.030
ASAT, *U/L*	836	[276;1466]	389	[188; 352]	0.188
ALAT, *U/L*	671	[214; 1065]	322	[168; 440]	0.256
Albumin, *g/L*	32	(4)	33	(6)	0.423
Prothrombin Time, *%*	72	(12)	88	(13)	0.007
Cholinesterase, *kU/L*	4.4	(0.8)	6.1	(1.5)	0.002
Platelets. *G/dL*	169	(48)	196	(33)	0.170
vWF, *%*	360	[325; 412]	325	[290; 333]	0.165
RiCo*, %*	433	(128)	317	(85)	0.030
Fibrinogen, *mg/dL*	370	(94)	538	(125)	0.002

Summary characteristics of postoperative maximum liver specific laboratory concentration during the first five days after surgery. Measurements are presented medians [25th percentile, 75th percentile]. All *P*-values are for Mann-Whitney-U tests. *ASAT, aspartate aminotransaminase; ALAT, alanine aminotransaminase; vWF, von Willebrand factor, RiCo, Ristocetin cofactor*

### Liver volumetric

Preoperative liver volume did not differ between the stratified groups. Resected liver volume as well as tumor volume was significantly bigger in patients with ∆HVPG was ≥1 mmHg as compared to patients with ∆HVPG ≤0 mmHg. The remaining postoperative liver volume was significantly smaller in patients with ∆HVPG was ≥1 mmHg. (Table 2).

### Catecholamines

**Fig. 1:**
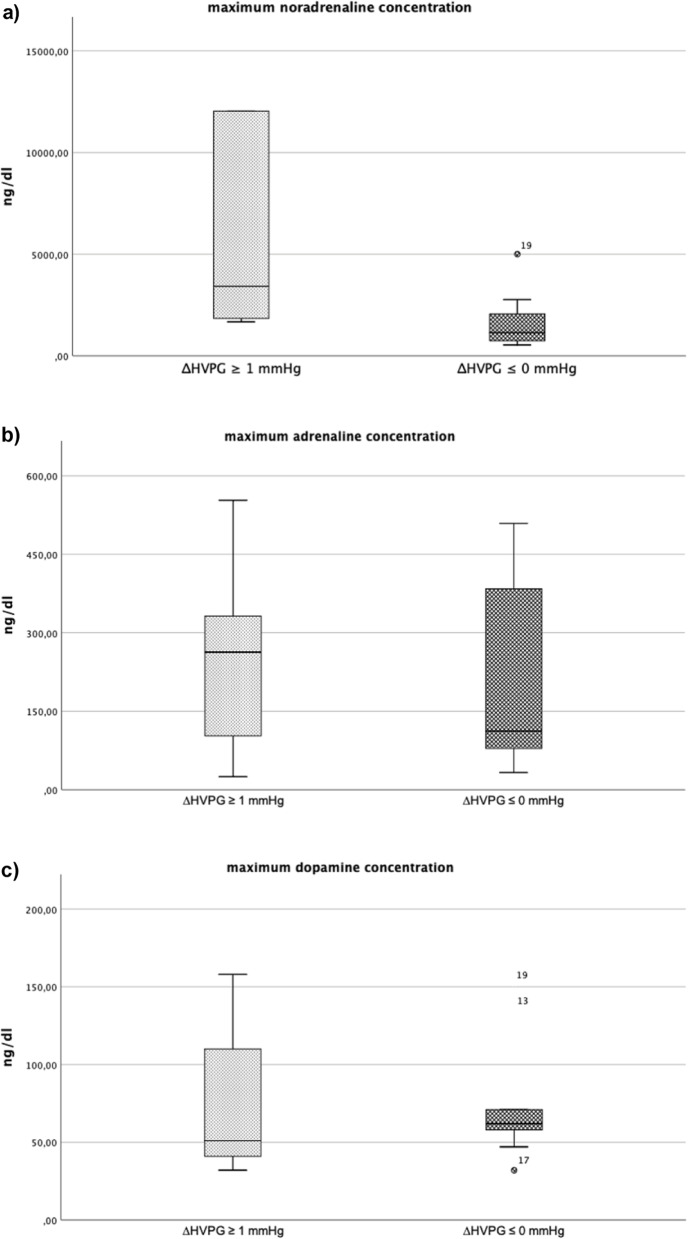
**a-c** The following plots show boxplot of the maximum (a) noradrenaline (b) adrenaline and (c) dopamine concentration between patients with ∆HVPG ≥1 mmHg as compared to patients with ∆HVPG ≤0 mmHg

Maximum postoperative noradrenalin concentration was significantly higher in patients with ∆HVPG was ≥1 mmHg as compared to patients with ∆HVPG ≤0 mmHg (3534 [2379; 19,679] vs 1376 [803; 2953], *p* <  0.009). Postoperative maximum adrenalin concentration (258 [66; 443] vs 129 [85; 443], *p* = 0.568) and postoperative maximum dopamine concentration (54 [43; 134] vs 62 [50;69], *p* = 0.653) remained similar between both groups. (Table 3) (Fig. 1a – c).

### Renin-aldosterone-angiotensin-system

**Fig. 2:**
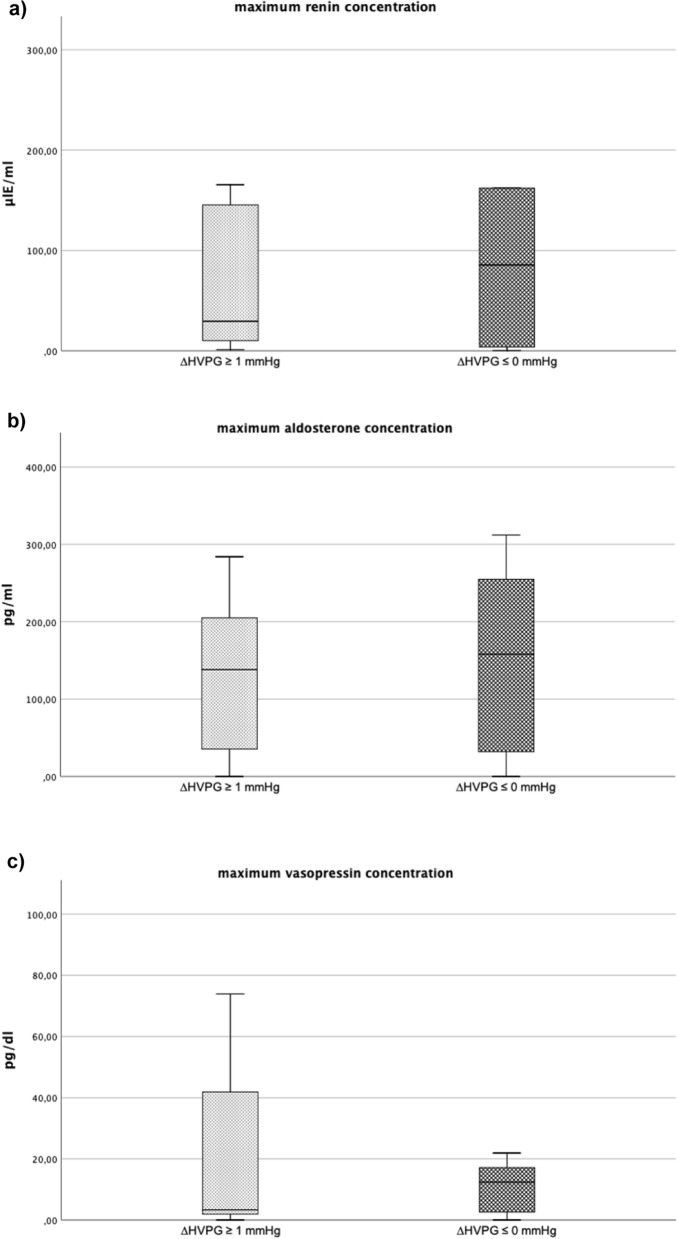
**a-c** The following plots show boxplot of the maximum (a) renin (b) aldosterone and (c) vasopressin concentration between patients with ∆HVPG ≥1 mmHg as compared to patients with ∆HVPG ≤0 mmHg

There were no significant differences in RAAS specific biomarkers, such as renin, aldosterone and vasopressin between both study groups. (Table 3). (Fig. 2a – c).

### Acute kidney injury

There were no significant differences in renin, aldosterone, vasopressin, noradrenalin, adrenalin and dopamine concentrations or liver volumetrics between patients with or without AKI. (Table 5).

**Table 5:** Data presented as median [25th percentile, 75th percentile]. All p-values are for Mann-Whitney-U tests. Pre-OP, preoperative, Post-OP, postoperative

			AKI (n = 9)		No AKI (n = 12)		*p -* Value
*Hepatic Venous Pressure Gradient*							
	Preoperative, *mmHg*		3.0	[2.0; 7.0]	4.0	[2.0; 6.3]	0.912
	Postoperative, *mmHg*		6.0	[4.0; 8.0]	5.0	[3.5; 7.0]	0,702
	∆HVPG, *mmHg*		2.0	[1.0; 3.0]	0.0	[0.0; 1.3]	0.247
*Renin-angiotensin-aldosterone-system*							
	Renin, *μlE/mL*	Baseline	12.7	[1.9; 73.6]	6.2	[2.5; 41.0]	0.321
		Max	31.3	[13.2; 431.1]	50.1	[5.6; 104.8|	0.481
	Aldosterone, *pg/mL*	Baseline	0	[0; 33.3]	16.5	[0; 48]	0.536
		Max	205	[159.8; 282.0]	40.5	[30.3; 167.8]	0.069
	Vasopressin, *pg/dL*	Baseline	0	[0; 6.1]	< 1	[< 1; 1]	0.918
		Max	7.6	[2.6; 26.9]	9.1	[1.6; 16.1]	0.696
*Sympathetic Nervous System*							
	Noradrenalin, *ng/dL*	Baseline	262	[195; 316]	270	[193; 744]	0.791
		Max	3416	[2605; 6759]	1750	[1070; 2416]	0.129
	Adrenalin, *ng/dL*	Baseline	10	[6; 25]	24	[7; 60]	0.591
		Max	239	[68; 270]	145	[103; 423]	0.717
	Dopamine, *ng/dL*	Baseline	3	[0; 10]	0	[0; 15]	0.884
		Max	79	[44; 148]	61	[51; 68]	0.717
*CT Liver Volumetrics*							
	Pre-OP Liver Volume, *mL*		1733	[1388; 2775]	1564	[1422; 1646]	0.696
	Resected Liver Volume, *mL*		262	[193; 618]	163	[81; 475]	0.315
	Post-OP Liver Volume, *mL*		1097	[841; 1518]	1151	[1009; 1393]	0.965
	Tumor Volume, *mL*		66	[24; 419]	24	[8; 219]	0.515

## Discussion

Based on our results it seems likely that the incidence of postoperative AKI after liver resection is higher in patients with increasing ∆HVPG (at least ≥1 mmHg) as compared to patients with unchanged or decreasing ∆HVPG.

58% of patients with ∆HVPG ≥1 mmHg developed AKI in contrast to only 22% of patients with stable ∆HVPG ≤0 mmHg. The reported incidence of AKI in an unselected patient population undergoing noncardiac general surgery is approximately 10% and thus, considerably lower than in our study population [1]. Although the incidence of AKI did not differ significantly between both study groups, serum creatinine was significantly higher in patients with a ∆HVPG ≥1 mmHg after surgery and their postoperative creatinine values remained significantly higher.

A retrospective analysis in over 38,000 patients undergoing noncardiac surgery showed that even minor increases in serum creatinine values are associated with a two-fold increased risk of death [1]. Our data highlight that patients undergoing liver resection have an increased risk of developing impaired kidney function, which has been associated with postoperative complications. Therefore, postoperative kidney function should be closely monitored in these patient population.

Furthermore, optimal intraoperative fluid management is important to maintain perioperative kidney function. Restrictive fluid therapy during abdominal surgery was associated with an increased risk of acute kidney injury compared to a liberal fluid regimen [12]. In our trial we performed esophagus doppler guided goal-directed fluid management to optimize intraoperative volume status and to minimize the risk of hypovolemia. All intraoperative hemodynamic parameters including MAP, stroke volume and cardiac output were similar between patients with increasing ∆HVPG ≥1 mmHg and patients with stable ∆HVPG ≤0 mmHg. A potential bias related to differences in intraoperative fluid management on the incidence of postoperative AKI can therefore be excluded.

It is very well known that intraoperative significant hypotension, which is defined as a mean arterial pressure < 65 mmHg, significantly increases the risk of myocardial ischemia and acute kidney injury.^2^ We controlled intraoperative blood pressure which has resulted in a MAP of 74 mmHg, therefore it seems very unlikely that intraoperative blood pressure did affect our results. We anesthetized all of our patients using volatile anesthetics. A previous study has shown that patients undergoing kidney transplantation had higher KIM-1 and NGAL values when sevoflurane was used.^3^ However, there was no significant effect in creatinine concentration during the first 8 days after kidney transplantation between using total intravenous anesthesia and volatile based anesthesia.^3^ Therefore, we can rule that the type of anesthesia has influenced our results.

Interestingly, a retrospective analysis of 167 patients undergoing living liver donor hepatectomy showed no effect of surgery on postoperative serum creatinine and BUN concentrations within the first three postoperative days [13]. This could be explained by apparent differences between both study populations, since we included patients scheduled for liver resection due to malignant liver disease who often suffered from preexisting comorbidities, while the aforementioned retrospective study only enrolled healthy middle-aged patients scheduled for liver donation. The abnormal liver parenchyma in patients with liver disease-associated liver cancer likely further enhance (postoperative) intrahepatic vascular resistance. The acute reduction of hepatic vascular bed due to liver resection results in increased hepatic vascular resistance and lead simultaneously to a reduction of hepatic vascular compliance [14]. The diseased liver parenchyma combined with acute perturbation of hepatoportal hemodynamics caused by surgery might be main trigger factors for an increase in postoperative HVPG. In patients with liver metastasis or cirrhosis, arterial hepatic blood flow was significantly higher as compared to healthy volunteers [15]. This might be a sign of decreased hepatic vascular compliance in malignant liver diseases. Therefore, it seems likely that reduced vascular compliance in these patients undergoing liver resection results in an increased postoperative HVPG which ultimately affects kidney function.

A portal venous pressure exceeding 5 mmHg defines portal hypertension [16]. Although, the median increase in patients with increasing ∆HVPG was only 2 mmHg, the incidence of postoperative AKI was up to 58%. Patients undergoing liver resection due to malignant liver disease might, however, be more vulnerable to even small increases in HVPG, specifically when HVPG rises abruptly. Based on our results we encourage tight perioperative kidney monitoring to detect deterioration of kidney function very early, specifically in patients undergoing liver surgery. This might help to initiate early treatment to prevent further deterioration which finally might improve postoperative outcome.

Increased portal venous pressure activates the hepatorenal reflex resulting in an impairment of kidney physiology [17]. The activity of the SNS plays an important role in early and late hepatorenal disorders [18]. In patients with cirrhosis, increased plasma noradrenalin concentrations are common, which is explained by the enhanced SNS activity [19]. Interestingly, in our study only noradrenalin concentrations were significantly higher in patients with ∆HVPG ≥1 mmHg as compared in patients with ∆HVPG ≤0 mmHg. Adrenaline and dopamine were not affected by postoperative ∆HVPG. While several trials registered higher RAAS activity in patients with liver cirrhosis [20], we did not observe difference in RAAS activity between patients with increasing vs. stable postoperative HVPG. An potential explanation might be that the RAAS is more active in patients with long-standing cirrhosis with pronounced portal hypertension [21]. Consequently, RAAS specific biomarkers might be unable to assess the hepatorenal reflex in the immediate postoperative period. Noradrenalin on the other hand is released immediately from sympathetic nerve endings during stressful events and might therefore be more appropriate to assess the hepatorenal reflex and might further reflect systemic stress.

Preoperative estimation of functional liver reserve is important to predict postoperative liver failure in patients undergoing liver resection surgery [22, 23]. Low Platelet count, decreased cholinesterase and increased bilirubin are strong predictors for impaired liver recovery after surgery [22, 23]. Estimated liver function in patients with increasing ∆HVPG ≥1 mmHg was significantly impaired and thus, these patients might be not able to compensate liver function after resection and counteract with an enhanced release of catecholamines, specifically noradrenalin. Noradrenaline is a strong alpha-receptor vasoconstrictor affecting kidney blood flow [24]. Therefore, this could be another explanation for the higher incidence of AKI in patients with ∆HVPG ≥1 mmHg.

A limitation of this trial is the small number of patients included and thus, the interpretation of our results has to be done with some caution. Although noradrenaline was significantly higher in patients with ∆HVPG ≥1 mmHg, catecholamines have a short half-life of only a few minutes. Elevated concentrations in our study were measured during surgery and within 2 h after surgery, therefor we cannot fully exclude the fact that this might be the stress response caused by surgery itself. In patients with ∆HVPG ≥1 mmHg the resected liver volume was significantly larger than in the other group, and we unfortunately do not have information of long-term changes in ∆HVPG.

In conclusion, our data demonstrate that patients undergoing liver resection who show an immediate postoperative increase in HVPG are at considerable risk to develop postoperative AKI and therefore kidney function should be monitored very closely. Due to our small sample size, our investigation should be seen as a hypothesis generating study. This emphasizes that further research is needed to clarify the effect of postoperative increases in HVPG on kidney function.

## Data Availability

The datasets generated and analyzed during the current study are available from the corresponding author on reasonable request.
